# Accuracy of Emergency Physicians in Grading Diastolic Dysfunction Using Visual Estimation of Waveforms

**DOI:** 10.5811/westjem.50527

**Published:** 2026-02-22

**Authors:** Daniel L. Puebla, Edward Lopez, Tarang Kheradia, Tony Zitek, Anthony Catapano, Robert A. Farrow, David H. Kinas

**Affiliations:** * Mount Sinai Medical Center, Department of Emergency Medicine, Miami Beach, Florida; † Florida International University, Herbert Wertheim College of Medicine, Miami, Florida; ‡ Loma Linda University, Department of Emergency Medicine, Loma Linda, California; § Kaiser Permanente Modesto Medical Center, Department of Emergency Medicine, Modesto, California

## Abstract

**Introduction:**

Diastolic dysfunction occurs when the ventricular walls of the heart stiffen and fail to relax appropriately. Early recognition in the emergency department (ED) enables identification of heart failure with preserved ejection fraction, guides antihypertensive and diuretic therapy, and facilitates timely cardiology referral to reduce morbidity and readmissions. Prior studies show emergency physicians (EP) can diagnose diastolic dysfunction with point-of-care ultrasound using mitral valve inflow velocities and tissue Doppler indices, although quantitative measurements are time-consuming. This study evaluates whether EPs can accurately diagnose and grade diastolic dysfunction based solely on visualization of mitral valve inflow velocities and tissue Doppler wave forms.

**Methods:**

After a focused training session, EPs (postgraduate year 1–3 residents, ultrasound fellows, and attendings) were randomized to review archived echocardiograms obtained by certified technicians. The EPs visually assessed echocardiograms for diastolic dysfunction (grades I–III) and whether they were considered “severe” (grade III). Their interpretations were then compared with a cardiologist’s gold-standard readings.

**Results:**

Twenty-three EPs interpreted 100 echocardiograms containing 25 of each grade. Overall accuracy for exact grading was 54.8%. Ultrasound attendings scored highest (70.0%), followed by non-ultrasound fellows (55.0%), attendings (54.0%), and residents (52.9%). For identification of any diastolic dysfunction, the EPs had a sensitivity of 84.6% (95% CI, 78.5–89.5%), specificity of 44.8% (95% CI, 31.7–58.5%), positive likelihood ratio (+LR) 1.53 (95% CI, 1.21–1.95), and negative likelihood ratio (-LR) 0.34 (95% CI, 0.22–0.54). For identification of severe diastolic dysfunction, the EPs’ intrepretations had a sensitivity of 59.4% (95% CI, 46.4–71.5%), specificity of 90.3% (95% CI, 85.0–94.3%), +LR 6.15 (95% CI 3.75–10.09), and -LR 0.45 (95% CI, 0.33–0.61).

**Conclusion:**

Emergency physicians can visually estimate diastolic function using mitral valve inflow velocities and tissue Doppler morphology with good sensitivity for detecting dysfunction and high specificity for identifying severe cases.

## INTRODUCTION

The number of cases of heart failure with preserved ejection fraction is rising every year. To diagnose this condition certain criteria must be met, among them symptoms of heart failure, an ejection fraction ≥ 50%, and evidence of diastolic dysfunction.^1^–^3^ In diastolic dysfunction the left ventricle is unable to relax and fill in diastole. Heart failure with preserved ejection fraction is diagnosed when the patient is symptomatic as a result of diastolic dysfunction.^2^ Diastolic dysfunction can provide useful prognostic information in patients with heart failure with reduced ejection fraction as well as in those suffering from sepsis; diastolic dysfunction is one of the strongest predictors of mortality in patients suffering from septic shock.^4^–^7^ Worsening diastolic dysfunction in patients with heart failure with preserved ejection fraction is also associated with increased mortality. Diagnosing diastolic dysfunction early provides the opportunity to treat contributory comorbid conditions before it becomes irreversible.^8^–^10^

It is hypothesized that diastolic dysfunction occurs due to systemic inflammation from other disease states, such as hypertension, diabetes, renal disease, and obesity, inducing structural and functional changes.^11^,^12^ As diastolic dysfunction worsens, the heart begins to remodel with increases in left ventricular thickness, hypertrophy of left ventricular mass, and left atrial enlargement.^13^ Typical treatments do not confer sufficient benefit when used in patients suffering from heart failure with preserved ejection fraction. However, early intervention and treatment of the comorbid conditions that contribute to heart failure with preserved ejection fraction may improve overall outcomes.^14^–^16^

Echocardiography is the most common modality to diagnose diastolic dysfunction. Two-dimensional echocardiography provides information on left atrial volume, left ventricular mass, mitral valve inflow velocity, and tissue Doppler indices, which are measurements used in diastolic evaluation.^16^ Emergency physicians (EP) can accurately diagnose diastolic dysfunction using point-of-care ultrasound (POCUS).^17^–^19^ Typical measurements in EP-performed POCUS include mitral valve inflow velocities measuring early diastolic filling (E) and late diastolic filling (A) velocities, as well as tissue Doppler indices measuring early diastolic mitral annular velocity (e′) and late diastolic mitral annular velocity (a′).^14^ Emergency physicians can accurately interpret tissue Doppler measurements and mitral valve inflow velocities.^18^,^20^ In patients with normal ejection fraction, the 2016 diastology guidelines of the American Society of Echocardiography require measurements of the E/e′ ratio, septal e′ or lateral e′ velocity, and tricuspid regurgitation as well as left atrial volume index. If > 50% of these measurements are positive, it is suggestive of diastolic dysfunction; 50% positive is suggestive of indeterminate dysfunction; and < 50% is suggestive of normal diastolic function.

However, the time needed to perform these studies, obtain measurements, and perform calculations may contribute to lack of adoption by emergency physicians.^21^,^22^ Visual estimation of mitral valve inflow velocities and tissue Doppler indices can expedite the time required to make diastolic dysfunction assessments and simplify the process in patients with heart failure with preserved ejection fraction. Prior studies have already demonstrated that emergency physicians can accurately assess cardiac dysfunction via visual estimation alone in left ventricular and right ventricular systolic function. To our knowledge, there have not been any studies evaluating the accuracy of performing diastolic assessment with only visual estimation.

**Population Health Research Capsule**

What do we already know about this issue?

*Diastolic dysfunction is rising in prevalence; obtaining the measurements to diagnose this condition can be time-consuming in the emergency department*.

What was the research question?

*Can emergency physicians reliably grade diastolic dysfunction using solely visual estimation of mitral valve inflow velocities and tissue Doppler indices?*

What was the major finding of the study?

*Their interpretations had a sensitivity of 84.6% (78.5*–*89.5%) for identifying diagnostic dysfunction and a specificity of 90.3% (85.0*–*94.3%) for severe dysfunction*.

How does this improve population health?

*Recognition enables identification of heart failure, guides antihypertensive and diuretic therapy, and facilitates early cardiology referral to reduce morbidity*.

We sought to determine whether EPs can accurately diagnose and grade diastolic dysfunction based solely on visualization of mitral valve inflow velocities and tissue Doppler waveforms. We hypothesized that their visual estimations would enable them to accurately diagnose diastolic dysfunction and grade of dysfunction.

## METHODS

This was a diagnostic accuracy study that compared EPs’ ability to visually estimate and grade diastolic cardiac function to that of a comprehensive cardiology echocardiogram. The study was performed in accordance with the Standards for Reporting of Diagnostic Accuracy guidelines. The study population consisted of 23 EPs who work in an academic hospital in Miami, FL, that has over 60,000 annual visits. The EPs had varying levels of training and experience; they included residents, ultrasound fellows who had performed < 1,000 ultrasounds, ultrasound fellowship-trained attendings, and attendings who were not ultrasound fellowship trained. They all participated voluntarily. The EPs attended a 30-minute didactic teaching session on the following: diastolic cardiac function; use of ultrasound to assess and interpret diastolic cardiac function; how to obtain and estimate these measurements; and how to visually estimate diastolic function based on mitral valve inflow velocities and septal tissue Doppler indices.

Ten tests, each containing 10 echocardiograms were created. Using a random number generator, subjects were then assigned diastolic studies on which they were tested. To ensure testing was standardized the examination was supervised, and participants were not allowed access to reference material. The primary study outcome was the accuracy of EP grading of diastolic dysfunction compared to a cardiologist’s interpretation. The secondary outcome was to identify whether EPs could accurately identify severe diastolic dysfunction (Grade III).

This study was approved by the Mount Sinai Medical Center of Florida Institutional Review Board.

Study investigators identified 1,746 echocardiograms performed by certified echocardiography technicians between February–April 2024 and extracted them from the electronic health record. These comprehensive cardiology studies were obtained from the main hospital and outpatient echocardiogram labs associated with the hospital. A total of 100 echocardiograms were selected for the purposes of testing: 25 contained grade 0 (normal) function; 25 contained grade I (impaired relaxation) dysfunction; 25 contained grade II (pseudo-normal) dysfunction; and 25 contained grade III (restrictive filling) dysfunction. Grade IV was not evaluated as this would have required a dynamic exam requiring that the patient perform the Valsalva maneuver to assess for irreversible restrictive pathology.

Exclusion criteria included ejection fraction < 50%, regional wall motion abnormality, mitral valve regurgitation or stenosis, mitral valve replacement, tachycardia, fusion of E and A waves, presence of pericardial effusion, and ventricular or atrial arrhythmias. With 75 positive and 25 negative cases, the study achieved strong statistical performance at an α = 0.05 significance level and power of 0.80, allowing detection of sensitivity of approximately 0.75. It also provided reasonable precision for estimating a specificity around 0.85, assuming a prevalence of diastolic dysfunction of 34.7%.^20^

We used cardiologist interpretation of the echocardiogram as the criterion standard. The EPs, who were blinded to the cardiology reports, were shown only images of the mitral valve inflow velocity and septal tissue Doppler waveforms. The EPs’ visual estimation responses were then compared to cardiology’s echocardiogram readings.

The method used for grading diastolic function was in accordance with the American Society for Echocardiography guidelines.^19^ After the didactics session, EPs were shown echocardiograms with the reports and measurements blinded to them. Study participants were asked to interpret mitral valve inflow velocities comparing the E wave to the A wave and tissue Doppler measurements and were asked to interpret septal E’ and septal A’. Both interpretations were done visually without measurements. The EPs were then asked to interpret these into their respective diastolic dysfunction grades. After collecting the data, we compared the accuracy of the EPs’ interpretation of the diastolic measurements to cardiology interpretation, which were done with measurements of E, A, septal E’, and septal A’ waves, respectively.

**Figure 1:**
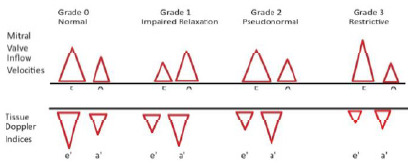
Figurative representation of a simplified grading system using mitral valve inflow velocities and tissue Doppler indices to determine degree of diastolic dysfunction.

Following is a simplified method for visual estimation of diastolic function of mitral valve inflow peak E velocity and peak A velocity, and septal mitral annular excursion velocities on tissue Doppler indices (E’ and A’) (Figure 1):

- Grade 0 (normal diastolic function): E > 80% of A and septal E’ > septal A’
- Grade I (impaired relaxation): E < 80% of A and septal E’ < septal A’
- Grade II (pseudo-normal): E > 80% of A and septal E’ < septal A’
- Grade III (restrictive filling): E > 2 times A and septal E’ < septal A’.

**Figure 2:**
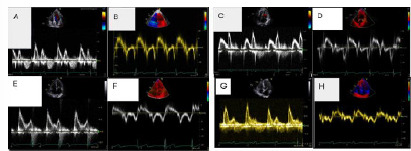
Demonstration of the mitral valve inflow velocities (MVI) and tissue Doppler indices (TDI) for respective grades of diastolic dysfunction. Image A and B are the MVI and TDI for a patient with grade 0 (normal diastolic function), image C and D for grade I, Image E and F for grade II, and image G and H for grade III.

Measurements were made on spectral Doppler tracings, and a standardized data form was used. This simplified grading method was developed on the patterns noted in the mitral valve inflow velocities and tissue Doppler waveforms noted in the respective diastolic grades (Figure 2).

The primary study outcome was emergency physician’s accuracy in measuring diastolic dysfunction compared to cardiology interpretation. We collected data on SurveyMonkey (Momentive Inc, San Mateo, CA), which we then converted on an Excel spreadsheet v16.98 (Microsoft Corporation, Redmond, WA). Sensitivity and specificity were calculated to measure whether EPs could diagnose diastolic dysfunction (of any grade) and identify grade III diastolic dysfunction.

## RESULTS

A total of 1,746 echocardiograms were obtained from the search. Patient demographic information is noted in the Table. In terms of diastolic dysfunction, the results showed that 1,377 echocardiograms (78.9%) demonstrated normal diastolic function; 204 (11.7%), grade I diastolic dysfunction; 134 (7.7%), grade II diastolic dysfunction; and 31 (1.8%) had grade III diastolic dysfunction.

A total of 23 physicians participated in the study, including two ultrasound fellowship-trained attendings, five attendings who were not ultrasound fellowship-trained, two ultrasound fellows, and 14 emergency medicine (EM) residents at different levels of training: three postgraduate year (PGY)-1 residents; six PGY-2; and five PGY-3. Eleven of the 14 EM residents had completed a four-week ultrasound rotation. Each physician assessed 10 echocardiograms for diastolic dysfunction, for a total of 230 assessments (from 100 unique echocardiograms). The overall mean score for identifying the exact grade of diastolic dysfunction was 54.8%, with individual scores ranging from 20%–80%. The scores varied according to the EPs’ level of experience and type of training. Ultrasound fellowship-trained attendings demonstrated a mean score of 70.0%, while non-ultrasound attendings had a mean score of 54.0%. Ultrasound fellows achieved a mean score of 55.0%, and EM residents had the lowest average score at 52.9%.

In terms of diagnostic performance for identifying diastolic dysfunction of any grade, the sensitivity was 84.6% (95% CI, 78.5–89.5%), while specificity was 44.8% (95% CI, 31.7–58.5%). The positive likelihood ratio (+LR) was calculated at 1.53 (95% CI, 1.21–1.95), and the negative likelihood ratio (-LR) was 0.34 (95% CI, 0.22–0.54). For the identification of severe diastolic dysfunction, sensitivity was 59.4% (95% CI, 46.4–71.5%), and specificity was 90.3% (95% CI: 85.0–94.3%). The +LR for severe dysfunction was 6.15 (95% CI, 3.75–10.09); and the -LR was 0.45 (95% CI, 0.33–0.61).

## DISCUSSION

Emergency physicians demonstrated varying degrees of accuracy in assessing grades of diastolic dysfunction, which was dependent on their level of ultrasound training. However, EPs demonstrated good sensitivity in diagnosing the presence of diastolic dysfunction and high specificity in identifying severe diastolic dysfunction using solely visual estimation of the diastolic waveforms.

A prior exploratory study performed by Del Rios et al demonstrated that EPs were able to obtain and identify mitral valve inflow velocities and tissue Doppler indices, achieving a high degree of consistency with cardiologist interpretations.^20^ Early identification of diastolic dysfunction of any grade in the ED can meaningfully alter a patient’s clinical course. Diastolic dysfunction is a powerful, independent predictor of incident heart failure and mortality. Recognizing it early at bedside enables risk-stratified disposition, expedited cardiology referral, and earlier disease-modifying care. Once diastolic dysfunction is recognized, aggressively addressing drivers such as hypertension improves outcomes.^8^ Importantly, left ventricular diastolic dysfunction may emerge before the classic signs are noted on electrocardiograms or blood tests show the troponin elevation associated with myocardial injury in patients presenting with chest pain.^23^

The range in scores in grading specific portions of diastolic dysfunction can be attributed to the overall lack of knowledge of diastology in EM. The didactic training in this study was 30 minutes, which may have not been enough for a group of EPs who knew very little about diastolic dysfunction assessment on echocardiogram. Therefore, educational interventions to enhance EM residents’ and non-ultrasound fellowship trained attendings’ understanding and diagnostic skills related to diastolic dysfunction would be beneficial in improving diagnostic accuracy in the ED. Incorporating education on diastolic dysfunction into the ultrasound curriculum during EM residency could help narrow this knowledge gap. In addition to further training, novel use of tools, such as artificial intelligence (AI), can assist with the grading of diastolic dysfunction, although their utility is dependent on the user’s skill and image input.^24^,^25^ While newer ultrasound systems incorporate AI-based, image-quality guidance, its impact on the accuracy of diastolic dysfunction grading remains uncertain.

The high sensitivity for identifying diastolic dysfunction and the high specificity for severe cases indicate that focused training could significantly improve diagnostic accuracy in clinical practice. The use of a visual grading system for diastolic dysfunction may prove useful in screening.

## LIMITATIONS

There are several limitations to this study. The first is that the echocardiograms were pre-acquired by echocardiography technicians and not the actual physician caring for the patient. Also, echocardiograms were excluded if the patient had any reduced ejection fraction, valvular abnormalities, or regional wall motion abnormalities. While this was done for study control, it could have limited the clinical applicability of this method and the diagnostic accuracy of the results. This study was focused on visual estimation of diastolic waveform patterns, which may be less accurate compared to the cardiologist’s criteria for grading diastolic dysfunction that includes other parameters.^21^ Our criterion standard comparison was that of cardiology echocardiography interpretation. Cardiology guidelines for this interpretation include measurements such as left atrial area, E/E’ ratio, E/A ratio, and more. Because EPs used only a portion of the current diastology guideline criteria due to the imposed time constraints of the ED, this reduced the accuracy of their overall interpretation compared to cardiology .

Furthermore, the positive likelihood ratio and negative likelihood ratio values for diastolic dysfunction and severe diastolic dysfunction fell short of ideal thresholds (≥ 10 and ≤ 0.1), with the positive likelihood ratio for diastolic dysfunction only 1.53 and the negative likelihood ratio 0.45. Length of training is a limitation as well. While a 30-minute didactic session may suffice as an introduction to this topic, longer training sessions would reinforce learning and improve overall accuracy of EPs’ recognition of diastolic dysfunction. A future study could evaluate retention of the diastology training.

Another potential limitation of this study is case selection bias, as the deliberate inclusion of equal numbers of diastolic dysfunction grades does not reflect real-world prevalence. This may limit generalizability. Additionally, although some echocardiograms were interpreted by multiple physicians, resulting in modest clustering by reader, this degree of correlation was minimal and unlikely to have meaningfully affected confidence intervals or conclusions.

## CONCLUSION

This study indicates that visual estimation demonstrates modest accuracy when differentiating between the grades of diastolic dysfunction. Grading of diastolic dysfunction is a useful skill with a myriad of applications in the ED, including for sepsis, heart failure, and fluid resuscitation, and it is important that it is included in the evaluation. Enhancing visual interpretation of diastolic dysfunction with AI measurements of diastolic dysfunction may also improve the emergency physician’s accuracy of this interpretation to safely implement this in the emergency department.

## Figures and Tables

**Table:** Characteristics of patients whose echocardiograms were selected for review in a study of emergency physicians’ ability to diagnose and grade diastolic dysfunction based solely on visualization.

Characteristic	Value
Mean age	58.4 ± 17.5
Race	
White	1,102 (63.17%)
Unknown/declined to state	286 (18%)
Black	129 (7.4%)
Other	111 (6.36%)
Multiracial	62 (3.5%)
Asian	21 (1.2%)
American Indian/Alaska Native	1 (0.05%)
Ethnicity	
Non-Hispanic	758 (43.4%)
Hispanic	668 (38.3%)
Unknown/decined to state	320 (18.3%)
Diastolic Dysfunction	
Normal diastolic function	1,377 (78.9%)
Grade I diastolic dysfunction	204 (11.7%)
Grade II diastolic dysfunction	134 (7.7%)
Grade III diastolic dysfunction	31 (1.8%)
